# Psychological Health and Diabetes Self-Management among Patients with Type 2 Diabetes during COVID-19 in the Southwest of Saudi Arabia

**DOI:** 10.3390/medicina58050675

**Published:** 2022-05-19

**Authors:** Abdulrhman H. Alkhormi, Mohamed Salih Mahfouz, Najim Z. Alshahrani, Abdulrahman Hummadi, Wali A. Hakami, Doha H. Alattas, Hassan Q. Alhafaf, Leena E. Kardly, Mulook A. Mashhoor

**Affiliations:** 1 Department of Preventive Medicine, King Fahd Central Hospital, Ministry of Health, Jazan 84211, Saudi Arabia; alkhormi1988@gmail.com; 2 Department of Family and Community Medicine, Faculty of Medicine, Jazan University, Jazan 82911, Saudi Arabia; 3 Department of Family and Community Medicine, College of Medicine, University of Jeddah, Jeddah 21589, Saudi Arabia; 4 Jazan Diabetes and Endocrine Center, Ministry of Health, Jazan 82723, Saudi Arabia; drhummadi@hotmai.com (A.H.); wali_733@hotmail.com (W.A.H.); dodod1450@gmail.com (D.H.A.); hassan19812005@gmail.com (H.Q.A.); lina1234387@gmail.com (L.E.K.); ashraf20091955@gmail.com (M.A.M.)

**Keywords:** psychological health, diabetes mellitus, self-care management, Saudi Arabia, COVID-19

## Abstract

*Background and objectives*: The prevalence of type 2 diabetes in Saudi Arabia is high and rising steeply. However, the management of type 2 diabetic patients has largely employed a medical approach and ignored the self-care management approach. This observation has even been obscured further by the COVID-19 pandemic, which has affected the psychological health of these patients. This study aimed to understand the effects of psychological health and DSM on type 2 diabetic patients in the Jazan region during COVID-19. *Materials and methods*: An analytical cross-sectional study was employed in this study. Participants were type 2 diabetic patients from the diabetic center at Jazan, Saudi Arabia. The Arabic-translated version of the Patient Health Questionnaire (PHQ-9) and Generalized Anxiety Disorder Scale (GAD-7) were used to collect data. Data were analyzed using SPSS software. *Results*: Depression and anxiety were higher in females compared to males and were more reported by participants from urban compared to rural settings. Smoking and Khat chewing were inappropriate diabetic self-care management practices while exercising was appropriate. A negative correlation was observed between depression vs. health care utilization, and depression vs. diabetic self-care management. Anxiety results also showed similar findings to that of depression. Additionally, depression and anxiety were easily predicted by urban residence, and diabetic self-care management was predicted by exercise. *Conclusions*: Adequate self-care behavior in patients with type 2 diabetes is needed. Medical professionals should ensure improved efforts to accurately ascertain how an individual can implement the recommended lifestyle changes and facilitate self-care education.

## 1. Introduction

Diabetes mellitus (DM), a multifactorial disease and most common chronic endocrine disorder, affects about 5–10% of adults globally [1]. If not diagnosed or well treated, DM can lead to multiple chronic complications that can result in disability and death [1]. Diabetes mellitus is on the rise in developing countries, Saudi Arabia inclusive. It is estimated that by 2030, the prevalence of diabetes among those aged 20–79 years may increase to 7.7% [2]. According to the World Health Organization, the East Mediterranean region ranks second in the global diabetes prevalence. As far as the Gulf countries are concerned, the prevalence rates of type 2 diabetes mellitus stand at 25.7%, 16.1%, 21%, and 31.6% of the general population in Bahrain, Oman, Kuwait, and Saudi Arabia, respectively [1,2].

While it is known that psychological disorders aggravate chronic conditions, diabetes mellitus is not exceptional. Many psychological disorders worsen the DM conditions of many people. There has been an observed rise in psychological disorders among the Saudi population during the COVID-19 period. As COVID-19 affected people, many of the professionals focused on addressing the virus’s physiological effects [2]. Patients with chronic diseases were highly affected by COVID-19. They were to manage their illness and cope with the challenging issues that accompanied the new situation, like minimized access to healthcare providers and living in isolation at home.

COVID-19 is caused by a large number of highly diverse enveloped, positive-sense, and single-stranded RNA viruses [3]. After a pneumonia outbreak in Wuhan, human-to-human contact was identified as a possible mode of transmission for COVID-19, and the WHO declared it a pandemic virus on 11 March 2019 [4]. The Saudi Arabian index case was discovered on 2 March 2020, immediately followed by a dramatic increase in confirmed cases [5].

The COVID-19 most commonly reported triggers of psychological disorders have been: lack of knowledge, fear, worry and concern, family member or friends’ infection, death, lockdown restrictions, quarantine, and confirmed or suspected COVID-19 infection [2]. Quarantine methods also led to a significant meltdown in economic growth, an increase in unemployment or financial insecurity, a rise in the standard of living, and significant reductions in government expenditure [6]. During this period, the commonly reported psychological disorders were depression, anxiety, and stress [2]; the prevalence of depression and anxiety stood at 33.7% and 31.9%, respectively [7].

Psychological disorder is the second most common cause of loss of disability-related life years and the third leading cause of disability-adjusted life years (DALY). There are two negative side effects of depression on the patients themselves and society: less active life and increased loss of productivity [8]. Indeed, depression is a significant issue for diabetic patients.

Approximately 15% of diabetic patients have major depression, whereas 9% of the general population have depression [9]. Diabetes mellitus and its effects generally have a high national economic burden, as it increases healthcare costs [10]. As such, effective management approaches are needed. The management of diabetes is lifelong and multidimensional, focusing majorly on controlling blood sugar levels [10]. Although medical management is key in DM management, the patients’ self-care management is also highly important; it is the key to better health and contributes to approximately 95% of DM management [10].

Self-care management is defined as people engaging to improve their health, prevent disease, limit activities to improve their own health, limit illness, and regain health [11]. Self-care-management is critical for glycemic control. Poor glycemic control is associated with poor self-management. Self-management consists of various components, including adhering to the diet pattern, performing routine blood glucose tests, engaging in regular physical activity, utilizing health centers, and achieving a favorable overall rating for diabetes self-management [10,12].

The female gender, education level, age, and higher socioeconomic status have been reported to be significantly associated with proper self-care [13]. Therefore, it was important to understand how selfcare management would significantly affect the face of COVID-19, DM, and psychological health. So, the purpose of this study was to understand the effects of psychological health and DSM on type 2 diabetic patients in the Jazan region during COVID-19. This study was guided by the following objectives:

1. To determine the prevalence of depression, anxiety, and DSM across the sociodemographic characteristics.
2. To assess the correlation between psychological health and DSM.
3. To determine the factors that strongly predict depression, anxiety, and DSM.

## 2. Materials and Methods

Design and setting: An analytical cross-sectional study was conducted in the diabetic center at Jazan, Saudi Arabia from 23 August 2021 to 2 February 2022. The diabetic center serves as a referral center for controlled and uncontrolled diabetic patients from all primary healthcare centers in the region. This center serves over 10,000 diabetic patients and it is the only center in Jazan.

Study population and sample size: Patients aged ≥ 18, diagnosed with type 2 diabetes for at least one year were included in this study. Patients with type 1 diabetes and mentally and severely ill were excluded from the study. The selection process was carried out to obtain a representative sample of type 2 diabetic patients. A sample size of 375 patients for this study was determined using the formula n = P (1 − P) *×* Zα^2^/d^2^, where n is the calculated sample size, P is the expected proportion in population, based on previous studies (the prevalence of mental illness among diabetic patients of 57% based on a study conducted in Jazan [14]), Z is the z-value for the selected level of confidence (95%), and d is absolute error or precision (0.05) [15].

The 5% precision catered for the nonresponse rate. Samples were enrolled using a systematic random sampling technique until the estimated sample size was achieved. This systematic sampling was dependent on the center’s medical records file numbers for the diabetic patients.

Data tool and collection: A participant self-administered questionnaire was used for the data collection. The Arabic-translated version of the Patient Health Questionnaire (PHQ-9) and Generalized Anxiety Disorder Scale (GAD-7) were used to collect data. These questionnaires used the 4-point Likert scale and were valid and reliable for determining the prevalence of depression and generalized anxiety disorder. The response on each item ranged from 0 “not at all”, 1 “several days”, 2 “more than half the days” and 3 “nearly every day”.

The PHQ-9 instrument consists of 9 items; each one is scored 0 to 3, yielding a severity score of 0 to 27. A score of 5, 10, 15, or 20 on the PHQ-9 scale indicates mild, moderate, moderately severe, or severe depression, respectively. A PHQ-9 score ≥ 10 was considered as depression [16].

The GAD-7 instrument consists of 7 items; each one is scored 0 to 3, yielding a severity score of 0 to 21. Scores of 5, 10, and 15 indicate mild, moderate, or severe anxiety, respectively. GAD-7 scores ≥ 10 were considered anxiety [17].

Additionally, data on diabetes self-management (DSMQ) was collected using the Arabic translation of the (DSMQ), whose validity and reliability were previously published by Schmitt et al. [18]. The DSMQ was divided into four subscales: glucose control (5 items: 1, 4, 6, 10, 12), dietary control (4 items: 2, 5, 9, 13), physical activity (3 items: 8, 11, 15), and healthcare utilization (3 items: 3, 7, 14). Each item was tracked among diabetic patients over the past 8 weeks using a 4-point scale (3: applies to me very much, 2: applies to me to a considerable degree; 1: applies to me to some degree; 0: does not apply to me).

The total Likert scores ranged from 0 to 10, with the higher score indicating better self-management. Based on the recommendation of Schmitt et al., the DSMQ scores of the participants were classified as “adequate” (scores that were greater than six) or “inadequate” (scores that were equal to or less than six).

Furthermore, the sociodemographic data of the participants were collected. These data included age, gender, level of education, residential place, marital status, occupation, social status, family monthly income, nationality, smoking status, khat chewing, exercise, and years since diabetes was diagnosed.

Data management and analysis: All the collected data were entered in an Excel sheet, cleaned, coded, and transported to SPSS version 26 for analysis. Descriptive statistics were conducted for all the coded variables and presented in a frequency table. The Chi-square test was used to test the statistical significance of associations between individual study variables and depression, anxiety, and DSM. Pearson’s correlation coefficient was used to evaluate the correlation between psychological health and diabetes self-management domains. Logistic regression models were used to assess depression, anxiety, and diabetes self-management predictors among type 2 diabetes mellitus. A *p*-value < 0.05 was used to indicate the statistically significant findings.

Ethical Consideration: Ethical approval was obtained from the Jazan Hospital Institutional Review Board (reference number: H-10-Z-073) with ethical approval number—2141. Written informed consent was obtained from all the participants before enrolment. For purposes of confidentiality, the questionnaires were preidentified to create anonymity. This study did not pose any risk of harm to any of the participants.

## 3. Results

As shown in Table 1, the majority of participants were females (51.7%) and between 50 and 65 years. Most of the participants were Saudi, married, and living in urban areas, at 89.6%, 65.9%, and 63.5%, respectively. Regarding education level and family income, 33.1% of patients were highly educated, and 46.5% had a monthly family income of less than 5000 SAR. Smoking and Khat chewing habits were prevalent among 18.7% and 20.0% of the study participants, respectively. The majority (38.1%) of the participants reported living with type 2 DM for 1–5 years, while 52.8% of the participants reported exercising.

As shown in Table 2, When the groups under each variable were compared with depression status to see whether there was a statistically significant difference between them using Chi-square, females had a statistically higher prevalence of depression compared to their male counterparts (*p* = 0.000). Participants between the ages of 20 and 34 were found to have a statistically significant higher rate of depression compared to those in other age groups (*p* = 0.033). Urban residents had a statistically significant higher rate of depression than rural residents (*p* = 0.007). Unemployed individuals had a statistically significant higher rate of depression than other groups (*p* = 0.008). Participants who exercised had a statistically significantly higher rate of depression compared to those that did not.

There were no statistically significant associations between depression and marital status, level of education, duration of diagnosis of diabetes mellitus, smoking, or khat chewing.

Table 3 demonstrates the distribution and Chi-square tests of anxiety in participants with type 2 diabetes mellitus (T2DM) according to their demographic characteristics (*n* = 375). Females had a higher prevalence of anxiety (65.6%) than males, a highly significant difference (*p* < 0.001). The rate of anxiety was higher in urban areas (60.6%) than in rural areas (*p* = 0.022). There were no statistically significant differences in other sociodemographic characteristics.

Table 4 demonstrates the distribution of diabetes self-management among participants with type 2 diabetes mellitus (T2DM) according to their demographic characteristics (*n* = 375). Smokers had a statistically significantly higher rate of inappropriate self-care behavior than nonsmokers (*p* = 0.018). Those who chewed Khat had a statistically significantly higher inappropriate self-care behavior than those who did not (*p* = 0.043). The participants who exercised had statistically significantly higher appropriate DSM compared to those that did not exercise (*p* = 0.008).

There were no statistically significant differences in other sociodemographic characteristics.

Table 5 demonstrates the correlation coefficient (Pearson’s r) between depression, anxiety, and the DSM subscale. The findings indicate that depression had a statistically significant negative correlation with healthcare utilization (r = 0.164, *p* < 0.01) and overall DSM score (r = 0.166, *p* < 0.001), yet with a positive statistically significant correlation with anxiety (r = 0.781, *p* < 0.01). Besides that, anxiety had a significant negative correlation with healthcare utilization (r = 0.180, *p* < 0.01) and overall DSM score (r = 0.173, *p* < 0.01).

On the other hand, the correlation coefficient between the DSM shows that glucose management had a significant positive correlation with dietary control (r = 0.557, *p* < 0.01). Furthermore, healthcare utilization had a significant positive correlation with dietary control, glucose management, and physical activity ((r = 0.429, *p* < 0.01), (r = 0.38, *p* < 0.014), and (r = 0.354, *p* < 0.01), respectively).

Table 6 shows the simple logistic regression analysis of factors associated with depression, anxiety, and DSM. In terms of depression, the analysis showed that urban residency is a statistically strong predictor of depression risk (OR = 1.78, *p* = 0.007, 95% C. I: 1.170–2.739). The rest of the sociodemographic characteristics were lesser predictors of depression. According to anxiety, the analysis established that urban residency was still found to be a statistically strong predictor of anxiety risk (OR = 1.644, *p* = 0.022, 95% C. I: 1.073–2.519).

The rest of the sociodemographics were lesser predictors of the characteristics of anxiety. Regarding the factors contributing to successful DSM, the analysis determined that exercise was a strong predictor of effective self-care (OR = 1.775, *p* = 0.008, 95% C. I: 1.161–2.712). On the other hand, smoking and Khat chewing were found to be less predictors of effective self-diabetic care, with (OR = 0.498, *p* = 0.019, 95% C. I: 0.278–0.893) and (OR = 0.567, *p* = 0.045, 95% C. I: 0.326–0.988), respectively.

## 4. Discussion

To the best of our knowledge, this is the first study to examine the prevalence of depression, anxiety, and diabetes self-management among patients with type 2 diabetes in Jazan, Saudi Arabia, during the COVID-19 pandemic. Regarding psychological health, we discovered a high prevalence of depression and anxiety among people with type 2 diabetes during the COVID-19 pandemic. Depressive and anxiety symptoms were reported at 54.4% and 47.1%, respectively, of all participants in this study. This rate is nearly identical to what was observed in Saudi Arabia and the Gulf countries during the COVID-19 outbreak among diabetic and nondiabetic patients [19].

Moreover, our findings corroborate what was discovered during the COVID-19 pandemic among diabetic patients in Brazil [20]; 43.3% of participants had anxiety while 45.1% of participants had depression. Our study discovered that depression rates were likewise similar to those observed in Bangladeshi patients with chronic diseases [21]; 59% of participants had anxiety while 71.6% of participants had depression.

The rate of depression and anxiety in Germany after the initial COVID-19 outbreak was 21.3% and 22.9%, respectively [22]. A subsequent Germany study reported the rates to be 19.3% for depression and 22.6% for anxiety [23]. These reported rates are lower than the rate observed in our study. The disparity between our population and other populations in other studies could be due to the inability to access healthcare during a lockdown. Social isolation and fear of infection have a negative effect on mental health or may result from other factors such as genetics, culture, or education. So, to avoid such unfavorable consequences, existing initiatives, including telephone consultations and online support services for mentally ill people who are experiencing the effects of the ongoing pandemic, could be used [24].

We discovered that women were more likely to suffer from diabetes-related depression and anxiety. Our results agree with those of past studies [25,26,27,28]. This observation is largely attributed to hormonal factors contributing to an increased risk of developing depression and anxiety. Pregnancy, fertility, menopause, and menstrual cycle issues increase women’s risk of developing depression and anxiety [29].

Subsequently, our study showed that residents of urban areas were more likely to suffer from depression and anxiety. This observation is similar to studies elsewhere [30,31,32]. Urbanization has several negative effects on mental health, including increased stressors and factors such as a crowded and polluted environment, a high level of violence, and decreased social support [33]. These factors can either increase or lead to depression and anxiety among the population.

Additionally, it was discovered that unemployed individuals suffer from depression, which is coherent with the findings of other studies [34,35]. Evidence shows that the majority of unemployed people are not able to attain the recommended diabetic self-care. Indeed, the high unemployment (at 48%) and poverty in the population studied have played an important role in the study findings.

Mental health decline has been partly attributed to the social consequences of pandemic restrictions and economic uncertainty, job loss, and unemployment. With the economic costs yet to be fully realized, those bearing the brunt will likely experience further deterioration in mental health [36]. Regular exercise is associated with a decreased risk of developing psychological problems such as depression.

Our findings indicate that individuals with type 2 diabetes who do not engage in regular physical activity are more likely to suffer from depression. This finding is consistent with previous studies [37,38].

According to diabetes self-management, such activities include glucose monitoring, adherence to prescribed medications and diets, regular healthcare follow-up, and participation in other physical activities [10]. This study’s findings indicate that two-thirds of Jazan’s population has poor self-care behaviors for diabetes management, with a rate of 69.9%. Thus, only 38.1% of the participants demonstrated adequate self-care behaviors. This observation is consistent with the findings of previous studies [30,31,32,33,34,35,36,37,38,39,40,41,42]. However, the percentage was higher in our findings than in these previous studies. This disparity could be due to many Saudis consuming honey and dates promoted by the holy Koran (considered sacred foods). They may believe that honey and dates can cure a variety of diseases that modern medicine is incapable of treating, yet this could be why they are unable to engage in physical activities during the lockdown. In addition, insufficient education about the disease and their lack of experience plays an active role in its management, leading to a lack of family support or inability to obtain a blood glucose monitoring device [43].

This disconnect between patient-perceived and self-reported self-care behaviors and unfilled glycemic outcomes should alert physicians to be more cautious in their advice to patients regarding self-care behavior. The current study discovered that diabetic patients who chewed Khat and smoked were more likely to have poor self-care for their diabetes. Our findings were consistent with previous studies [44,45,46]. There is a relationship between the habit of Khat chewing and the development of noninsulin-dependent diabetes mellitus, which may be explained by the adverse health effects of pesticide residues on Khat chewers. Khat chewers usually have significantly lower insulin than nonchewers because chewing Khat elevates cortisol levels and lowers insulin secretion and sensitivity [47].

Diabetes self-care requires a high level of problem-solving ability, and both depression and anxiety have a negative impact on this ability. This can lead to poorer self-care practices and ultimately poorer glycemic control. The current study demonstrates a negative correlation between depression and healthcare utilization, as well as between depression and DSM. As healthcare utilization and diabetes self-care management decreased, depression increased.

Similarly, anxiety increased with decreasing healthcare utilization and DSM. These findings were consistent with previous studies [48,49,50] that found healthcare utilization and DSM to be important considerations in addressing depression and anxiety among diabetic patients. Healthcare utilization, dietary control, glucose management, and physical activity positively correlated with DSM. These aspects are components of the diabetes self-care management package [10], and in this study, a satisfactory demonstration of their usability was observed.

Urban residence strongly predicted both anxiety and depression. Participants who lived in urban settings were more likely to develop anxiety and/or depression. This observation is explained by the social setup of the urban settings, because it is characterized by violence, congestion/crowding, and much stressful daily needs, compared to their counterparts in the rural settings.

## 5. Limitation

This study has several limitations. First, the current study was conducted in a single city, which cannot be considered representative of the entire Saudi population. Second, DSMQ, PHQ-9, and GAD-7 were evaluated purely based on participant self-reporting. As a result, the data may contain bias due to either over- or under-reporting. Additionally, this study did not cater for data about diabetes control, body weight, and dietary habits. As such, we do not know if patients were in proper glycemic control or not, normal weight, overweight or obese, or followed proper food intake or not. Lastly, HbA1c was not measured, yet it correlates with the risk of long-term diabetes complications.

## 6. Conclusions

Depression and anxiety are prevalent among the studied type 2 diabetic patients. The female gender and urban residents are associated with depression and anxiety in patients with type 2 diabetes. Diabetic patients should be screened for signs and symptoms of depression and should be referred to a social worker or psychologist regularly. Additionally, poor self-care behavior is widely prevalent among patients with type 2 DM in Jazan, Saudi Arabia. Smokers and Khat chewers are significantly associated with inadequate self-care behavior.

It is, therefore, critical to ensure adequate diabetes self-care behavior in patients with type 2 DM. Medical professionals should ensure ongoing efforts to accurately ascertain how individuals can implement the recommended lifestyle changes and raise self-care education.

## Figures and Tables

**Table 1:** Demographic characteristics of participants (*n* = 375).

Variable	Category	Number (%)
Gender	Male Female	181(48.3%) 194(51.7%)
Age in years	20–34 35–49 50–64 >65	21(5.6%) 115(30.7%) 156(41.6%) 83(22.1%)
Residence	Rural Urban	137(36.5%) 238(63.5%)
Education	Illiterate Writes and Reads Elementary Intermediate Secondary High education	73(19.5%) 28(7.5%) 45(12%) 39(10.4%) 66(17.6%) 124(33.1)
Occupation	Unemployed Farmer Business Public sector Private sector	180(48%) 12(3.2%) 18(4.8%) 132(35.2%) 33(8.8%)
Marital status	Single Married Divorced Widowed	60(16%) 247(65.9%) 22(5.9%) 46(12.3%)
Family income (SAR/month)	Less than 5000 From (5000–9999) From (10,000–14,999) More than 15,000	171(45.6%) 104(27.7%) 54(14.4%) 46(12.3%)
Nationality	Saudi Non-Saudi	336(89.6%) 39(10.4%)
Exercise	Yes No	198(52.8%) 177(47.2%)
Smoking	Yes No	70(18.7%) 305(81.3)
Khat chewing	Yes No	75(20%) 300(80%)
Duration of diabetes	1–5 Years 6–10 Years 11–15 Years 16–20 Years More than 20	143(38.1%) 89(23.7%) 58(15.5%) 42(11.2%) 43(11.5%)

**Table 2:** Distribution of depression according to demographic characteristics of participants (*n* = 375).

Variable	Category	Normal	Moderate to Severe	*p*-Value
Gender	Female Male	67 (34.5%) 104 (57.5%)	127 (65.5%) 77 (42.5%)	**0.000**
Age	20–34 35–49 50–65 ≥65	7 (33.3%) 55 (47.8%) 81 (51.9%) 28 (33.7%)	14 (66.7%) 60 (52.2%) 75 (48.1%) 55 (66.3%)	**0.033**
Residence	Rural Urban	75 (54.7%) 96 (40.3%)	62 (45.3%) 142 (59.7%)	**0.007**
Education	Illiterate Reads and writes Elementary Intermediate Secondary Higher education	28 (38.4%) 14 (50.0%) 17 (37.8%) 21 (53.8%) 28 (42.4%) 63 (50.8%)	45 (61.6%) 14 (50.0%) 28 (62.2%) 18 (46.2%) 38 (57.6%) 61 (49.2%)	0.351
Occupation	Unemployed Farmer Business Public sector job Private sector job	66 (36.7%) 9 (75.0%) 9 (50.0%) 68 (51.5%) 19 (57.6%)	114 (63.3%) 3 (25.0%) 9 (50.0%) 64 (48.5%) 14 (42.4%)	**0.008**
Marital status	Single Married Divorce Widow	27 (45.0%) 122 (49.4%) 8 (36.4%) 14 (30.4%)	33 (55.0%) 125 (50.6%) 14 (63.6%) 32 (69.6%)	0.091
Family income (SR/Month)	<5000 From (5000–9999) From(10,000–14,999) >15,000	72 (42.1%) 53 (51.0%) 23 (42.6%) 23 (50.0%)	99 (57.9%) 51 (49.0%) 31 (57.4%) 23 (50.0%)	0.457
Nationality	Non-Saudi Saudi	22 (56.4%) 149 (44.3%)	17 (43.6%) 187 (55.7%)	0.152
Smoking	No Yes	142 (46.6%) 29 (41.4%)	163 (53.4%) 41 (58.6%)	0.437
Khat chewing	No Yes	134 (44.7%) 37 (49.3%)	166 (55.3%) 38 (50.7%)	0.486
Exercise	No Yes	71 (40.1%) 100 (50.5%)	106 (59.9%) 98 (45.5%)	**0.044**
Duration of diabetes	From 1–5 years From 6–10 years From 11–15 years From 16–20 years >20 years	71 (49.7%) 39 (43.8%) 22 (37.9%) 19 (45.2%) 20 (46.5%)	72 (50.3%) 50 (56.2%) 36 (62.1%) 23 (54.8%) 23 (53.5%)	0.653

Bold to indicate that is *p* < 0.05 (significant).

**Table 3:** Distribution of anxiety according to demographic characteristics of participants (*n* = 375).

Variable	Category	Normal	Moderate to Severe	*p*-Value
Gender	Female Male	87 (44.8%) 111 (61.3%)	107 (55.2%) 70 (38.7%)	**0.001**
Age	20–34 35–49 50–65 ≥65	7 (33.3%) 64 (55.7%) 85 (54.5%) 42 (50.6%)	14 (66.7%) 51 (44.3%) 71 (45.5%) 41 (49.4%)	0.272
Residence	Rural Urban	83 (60.6%) 115 (48.3%)	54 (39.4%) 123 (51.7%)	**0.022**
Education	Illiterate Reads and writes Elementary Intermediate Secondary Higher education	34 (46.6%) 15 (53.6%) 22 (48.9%) 21 (53.8%) 35 (53.0%) 71 (57.3%)	39 (53.4%) 13 (46.4%) 23 (51.1%) 18 (46.2%) 31 (47.0%) 53 (42.7%)	0.788
Occupation	Unemployed Farmer Business Public sector job Private sector job	87 (48.3%) 9 (75.0%) 12 (66.7%) 72 (54.5%) 18 (54.5%)	93 (51.7%) 3 (25.0%) 6 (33.3%) 60 (45.5%) 15 (45.5%)	0.248
Marital status	Single Married Divorce Widow	32 (53.3%) 136 (55.1%) 8 (36.4%) 22 (47.8%)	28 (46.7%) 111 (44.9%) 14 (63.6%) 24 (52.2%)	0.340
Family income (SR/Month)	<5000 From (5000–9999) From (10,000–14,999) >15,000	83 (48.5%) 58 (55.8%) 26 (48.1%) 31 (67.4%)	88 (51.5%) 46 (44.2%) 28 (51.9%) 15 (32.6%)	0.111
Nationality	Non-Saudi Saudi	23 (59.0%) 175 (52.1%)	16 (41.0%) 161 (47.9%)	0.415
Smoking	No Yes	158 (51.8%) 40 (57.1%)	147 (48.2%) 30 (42.9%)	0.420
Exercise	No Yes	86 (48.6%) 112 (56.6%)	91 (51.4%) 86 (43.4%)	0.122
Khat chewing	No Yes	157 (52.3%) 41 (54.7%)	143 (47.7%) 34 (45.3%)	0.717
Duration of diabetes	From 1–5 years From 6–10 years From 11–15 years From 16–20 years	83 (58.0%) 45 (50.6%) 27 (46.6%) 18 (42.9%) 25 (58.1%)	60 (42.0%) 44 (49.4%) 31 (53.4%) 24 (57.1%) 18 (41.9%)	0.306

Bold to indicate that is *p* < 0.05 (significant).

**Table 4:** Distribution of diabetes self-management according to the demography of participants (*n* = 375).

Variable	Category	Inappropriate	Appropriate	*p*-Value
Gender	Female Male	117 (60.3%) 115 (63.5%)	77 (39.7%) 66 (36.5%)	0.520
Age	20–34 35–49 50–65 ≥65	14 (66.7%) 74 (64.3%) 96 (61.5%) 48 (57.8%)	7 (33.3%) 41 (35.7%) 60 (38.5%) 35 (42.2%)	0.781
Residence	Rural Urban	85 (62.0%) 147 (61.8%)	52 (38.0%) 91 (38.2%)	0.957
Exercise	No Yes	122 (68.9%) 110 (55.6%)	55 (31.1%) 88 (44.4%)	**0.008**
Education	Illiterate Reads and writes Elementary Intermediate Secondary Higher education	39 (53.4%) 18 (64.3%) 28 (62.2%) 24 (61.5%) 46 (69.7%) 77 (62.1%)	34 (46.6%) 10 (35.7%) 17 (37.8%) 15 (38.5%) 20 (30.3%) 47 (37.9%)	0.550
Occupation	Unemployed Farmer Business Public sector job Private sector job	105 (58.3%) 7 (58.3%) 12 (66.7%) 85 (64.4%) 23 (69.7%)	75 (41.7%) 5 (41.7%) 6 (33.3%) 47 (35.6%) 10 (30.3%)	0.661
Marital status	Single Married Divorce Widow	38 (63.3%) 147 (59.5%) 17 (77.3%) 30 (65.2%)	22 (36.7%) 100 (40.5%) 5 (22.7%) 16 (34.8%)	0.382
Family income (SR/Month)	<5000 From (5000–9999) From (10,000–14,999) >15,000	110 (64.3%) 65 (62.5%) 29 (53.7%) 28 (60.9%)	61 (35.7%) 39 (37.5%) 25 (46.3%) 18 (39.1%)	0.572
Nationality	Non-Saudi Saudi	24 (61.5%) 208 (61.9%)	15 (38.5%) 128 (38.1%)	0.964
Smoking	No Yes	180 (59.0%) 52 (74.3%)	125 (41.0%) 18 (25.7%)	**0.018**
Khat chewing	No Yes	178 (59.3%) 54 (72.0%)	122 (40.7%) 21 (28.0%)	**0.043**
Duration of diabetes	From 1–5 years From 6–10 years From 11–15 years From 16–20 years >20 years	91 (63.6%) 49 (55.1%) 38 (65.5%) 25 (59.5%) 29 (67.4%)	52 (36.4%) 40 (44.9%) 20 (34.5%) 17 (40.5%) 14 (32.6%)	0.569

Bold to indicate that is *p* < 0.05 (significant).

**Table 5:** Correlation between psychological health and diabetes self-management among participants (*n* = 375).

	Variable	I	II	III	IV	V	VI	VII
I	Depression	1.00						
II	Anxiety	0.781 *	1.00					
III	Dietary control	−0.66	−0.089	1.00				
IV	Glucose management	0.42	0.028	0.557 *	1.00			
V	Physical activity	−0.047	−0.068	0.310 *	0.467 *	1.00		
VI	Healthcare use	−0.164 *	−0.180 *	0.429 *	0.384 *	0.354 *	1.00	
VII	Total—DSM	−0.166 *	−0.173 *	0.687 *	0.709*	0.646 *	0.716 *	1.00

* Correlation significant at 0.01 level (2-tailed).

**Table 6:** Logistic regression model predicting depression, anxiety, and diabetes self-management among the participants.

Variable	Categories	B	S. E	Wald	*p*-Value	OR	95% CI	
							lower	upper
			Depression					
Gender	Female * Male	- −0.940	- 0.213	- 19.465	- 0.000	- 0.391	- 0.257	- 0.593
Residence	Rural * Urban	- 0.82	- 0.217	- 7.215	*-* 0.007 ^$^	- 1.78	- 1.170	- 2.739
Age	20–34 * 35–49 50–64 >65	- 0.018 −0.588 −0.752	- 0.518 0.298 0.282	8.584 0.001 3.897 7.108	0.035 0.972 0.048 0.008	- 1.018 0.555 0.471	- 0.369 0.310 0.271	- 2.810 0.996 0.819
Occupation	Unemployed * Farmer Business Public sector Private sector	- −1.645 −0.547 −0.607 −0.852	- 0.684 0.496 0.233 0.385	13.24 5.779 1.214 6.795 4.904	0.010 0.016 0.271 0.009 0.027	- 0.193 0.579 0.545 0.427	- 0.050 0.219 0.345 0.201	- 0.738 1.531 0.860 0.907
Exercise	No * Yes	- −0.421	- 0.209	- 4.053	*-* 0.044	- 0.656	- 0.436	- 0.989
			Anxiety					
Gender	Female * Male	- −0.668	- 0.210	- 10.109	*-* 0.001	- 0.513	- 0.340	- 0.774
Residence	Rural * Urban	- 0.497	- 0.218	- 5.214	*-* 0.022 ^$^	- 1.644	- 1.073	- 2.519
			DSM					
Smoking	No * Yes	- −0.696	- 0.297	- 5.487	*-* 0.019	- 0.498	- 0.278	- 0.893
Khat chewing	No * Yes	- −0.567	- 0.283	- 4.017	*-* 0.045	- 0.567	- 0.326	- 0.988
Exercise	No * Yes	- 0.574	- 0.216	- 6.024	- 0.008 ^$^	- 1.775	- 1.161	- 2.712

* Reference group; OR: Odds ratio; CI: Confidence interval; ^$^ *p* < 0.05 (significant).

## Data Availability

Not applicable.

## References

[1] Abdullah M., Mansour A. (2020). The Prevalence and Risk Factors of Type 2 Diabetes Mellitus ( DMT2 ) in a Semi-Urban Saudi Population. Int. J. Environ. Res. Public Health.

[2] Alzahrani F., Sabah A.A. (2022). Prevalence and factors associated with mental health problems in Saudi general population during the coronavirus disease 2019 pandemic: A systematic review and meta-analysis. PsyCh J..

[3] Chan J.F.W., Kok K.H., Zhu Z., Chu H., To KK W., Yuan S., Yuen K.Y. (2020). Genomic characterization of the 2019 novel human-pathogenic coronavirus isolated from a patient with atypical pneumonia after visiting Wuhan. Emerg. Microbes Infect..

[4] He F., Deng Y., Li W. (2020). Coronavirus disease 2019: What we know?. J. Med. Virol..

[5] Alshahrani N.Z., Alshahrani S.M., Farag S., Rashid H. (2021). Domestic Saudi Arabian Travellers’ Understanding about COVID-19 and Its Vaccination. Vaccines.

[6] Braquehais M.D., Vargas-Cáceres S., Gómez-Durán E., Nieva G., Valero S., Casas M., Bruguera E. (2020). The impact of the COVID-19 pandemic on the mental health of healthcare professionals. QJM Int. J. Med..

[7] Alshahrani S.M., Alshahrani N.Z., Alakhali K.M. (2021). COVID-19 triggers the incidence of diabetic ketoacidosis: A case report of uncontrolled type 2 diabetes mellitus. Int. J. Pharm. Res..

[8] Alhunayni N.M., Mohamed A.E., Hammad S.M. (2020). Prevalence of depression among type-II diabetic patients attending the diabetic clinic at arar national guard primary health care center, Saudi Arabia. Psychiatry J..

[9] Fiest K.M., Jette N., Quan H., St Germaine-Smith C., Metcalfe A., Patten S.B., Beck C.A. (2014). Systematic review and assessment of validated case definitions for depression in administrative data. BMC Psychiatry.

[10] Alotaibi B.B. (2020). Self-Care Management Practices of Diabetic Patients Type 2 in Saudi Arabia. Open J. Nurs..

[11] Levin L.S., Idler E.L. (1983). Self-care in health. Annu. Rev. Public Health.

[12] Alshahrani S.M., Alzahran M., Alakhali K., Vigneshwaran E., Iqbal M.J., Khan N.A., Othman A., Al-Worafi Y., Alavudeen S.S. (2020). Association Between Diabetes Consequences and Quality of Life Among Patients With Diabetes Mellitus in the Aseer Province of Saudi Arabia. Open Access Maced. J. Med. Sci..

[13] Alyaemni A. (2019). Socio-demographic factors associated with diabetes self-care activities at a primary healthcare center in Riyadh: An analytical cross-sectional study. POJ Diabetes Obes..

[14] Albasheer O.B., Mahfouz M.S., Solan Y., Khan D.A., Muqri M.A., Almutairi H.A., Alahmed H.A. (2018). Depression and related risk factors among patients with type 2 diabetes mellitus, Jazan area, KSA: A cross-sectional study. Diabetes Metab.Syndr. Clin. Res. Rev..

[15] Charan J., Biswas T. (2013). How to calculate sample size for different study designs in medical research?. Indian J. Psychol. Med..

[16] Kroenke K., Spitzer R.L., Williams J.B. (2001). The PHQ-9: Validity of a brief depression severity measure. J. Gen. Intern. Med..

[17] Spitzer R.L., Kroenke K., Williams J.B., Löwe B. (2006). A brief measure for assessing generalized anxiety disorder: The GAD-7. Arch. Intern. Med..

[18] Schmitt A., Gahr A., Hermanns N., Kulzer B., Huber J., Haak T. (2013). The Diabetes Self-Management Questionnaire (DSMQ): Development and evaluation of an instrument to assess diabetes self-care activities associated with glycaemic control. Health Qual. Life Outcomes.

[19] Al-Sofiani M.E., Albunyan S., Alguwaihes A.M., Kalyani R.R., Golden S.H., Alfadda A. (2021). Determinants of mental health outcomes among people with and without diabetes during the COVID-19 outbreak in the Arab Gulf Region. J. Diabetes.

[20] Souza G.F.D.A., Praciano G.D.A.F., Ferreira O.D.C., Paiva M.C., Jesus R.P.F.S.D., Cordeiro A.L.N., Souza A.S.R. (2021). Factors associated with psychic symptomatology in diabetics during the COVID-19 pandemic. Rev. Bras. Saúde Matern. Infant..

[21] Sayeed A., Kundu S., Al Banna M., Christopher E., Hasan M.T., Rasheda Begum M., Islam Khan M.S. (2020). Mental health outcomes of adults with comorbidity and chronic diseases during the COVID-19 pandemic: A matched case-control study. Psychiatr. Danub..

[22] Moradian S., Teufel M., Jahre L., Musche V., Fink M., Dinse H., Bäuerle A. (2021). Mental health burden of patients with diabetes before and after the initial outbreak of COVID-19: Predictors of mental health impairment. BMC Public Health.

[23] Musche V., Kohler H., Bäuerle A., Schweda A., Weismüller B., Fink M., Skoda E.M. (2021). COVID-19-related fear, risk perception, and safety behavior in individuals with diabetes. Healthcare.

[24] Alshahrani N.Z., Alshahrani S.M., Alshahrani A.M., Leggat P.A., Rashid H. (2021). Compliance of the Gulf Cooperation Council airlines with COVID-19 mitigation measures. J. Travel Med..

[25] Sun N., Lou P., Shang Y., Zhang P., Wang J., Chang G., Shi C. (2016). Prevalence and determinants of depressive and anxiety symptoms in adults with type 2 diabetes in China: A cross-sectional study. BMJ Open.

[26] Alzahrani A., Alghamdi A., Alqarni T., Alshareef R., Alzahrani A. (2019). Prevalence and predictors of depression, anxiety, and stress symptoms among patients with type II diabetes attending primary healthcare centers in the western region of Saudi Arabia: A cross-sectional study. Int. J. Ment. Health Syst..

[27] Hussain S., Habib A., Singh A., Akhtar M., Najmi A.K. (2018). Prevalence of depression among type 2 diabetes mellitus patients in India: A meta-analysis. Psychiatry Res..

[28] El Mahalli A.A. (2015). Prevalence and predictors of depression among type 2 diabetes mellitus outpatients in Eastern Province, Saudi Arabia. Int. J. Health Sci..

[29] Zender R., Olshansky E. (2009). Women’s mental health: Depression and anxiety. Nurs. Clin..

[30] Niraula K., Kohrt B.A., Flora M.S., Thapa N., Mumu S.J., Pathak R., Shrestha R. (2013). Prevalence of depression and associated risk factors among persons with type-2 diabetes mellitus without a prior psychiatric history: A cross-sectional study in clinical settings in urban Nepal. BMC Psychiatry.

[31] Rahim S.I.A., Cederblad M. (1989). Epidemiology of mental disorders in young adults of a newly urbanized area in Khartoum, Sudan. Br. J. Psychiatry.

[32] Camara A., Baldé N.M., Enoru S., Bangoura J.S., Sobngwi E., Bonnet F. (2015). Prevalence of anxiety and depression among diabetic African patients in Guinea: Association with HbA1c levels. Diabetes Metab..

[33] Srivastava K. (2009). Urbanization and mental health. Ind. Psychiatry J..

[34] Mukrim M.E., Alshammari N.M., Alshammari W.M., Alshammari M.S., Alshammari Y.N., Alshammari A.S., Alshammari M.S. (2019). Prevalence of depression, anxiety, and stress among diabetes mellitus patients in Arar, Northern Saudi Arabia. Age.

[35] Park C.Y., Kim S.Y., Gil J.W., Park M.H., Park J.H., Kim Y. (2015). Depression among Korean adults with type 2 diabetes mellitus: Ansan-community-based epidemiological study. Osong Public Health Res. Perspect..

[36] Achdut N., Refaeli T. (2020). Unemployment and psychological distress among young people during the COVID-19 pandemic: Psychological resources and risk factors. Int. J. Environ. Res. Public Health.

[37] Rosenbaum S., Tiedemann A., Sherrington C., Curtis J., Ward P.B. (2014). Physical activity interventions for people with mental illness: A systematic review and meta-analysis. J. Clin. Psychiatry.

[38] Salinero-Fort M.A., Gómez-Campelo P., San Andrés-Rebollo F.J., Cárdenas-Valladolid J., Abánades-Herranz J.C., de Santa Pau E.C., De Burgos-Lunar C. (2018). Prevalence of depression in patients with type 2 diabetes mellitus in Spain (the DIADEMA Study): Results from the MADIABETES cohort. BMJ Open.

[39] Alshahri B.K., Bamashmoos M., Alnaimi M.I., Alsayil S., Basager S., Al-Hariri M.T., Doss C.A.V. (2020). Assessment of self-management care and glycated hemoglobin levels among type 2 diabetes mellitus patients: A cross-sectional study from the kingdom of Saudi Arabia. Cureus.

[40] Utli H., Doğru B.V. (2021). The effect of the COVID-19 pandemic on self-management in patients with type 2 diabetics. Prim. Care Diabetes.

[41] Sayeed K.A., Qayyum A., Jamshed F., Gill U., Usama S.M., Asghar K., Tahir A. (2020). Impact of diabetes-related self-management on glycemic control in type II diabetes mellitus. Cureus.

[42] Saad A.M., Younes Z.M., Ahmed H., Brown J.A., Al Owesie R.M., Hassoun A.A. (2018). Self-efficacy, self-care and glycemic control in Saudi Arabian patients with type 2 diabetes mellitus: A cross-sectional survey. Diabetes Res. Clin. Pract..

[43] Shi C., Zhu H., Liu J., Zhou J., Tang W. (2020). Barriers to Self-Management of Type 2 Diabetes during COVID-19 Medical Isolation: A Qualitative Study. Diabetes Metab. Syndr. Obes. Targets Ther..

[44] Saghir S.A., Alhariri A.E., Alkubat S.A., Almiamn A.A., Aladaileh S.H., Alyousefi N.A. (2019). Factors associated with poor glycemic control among type-2 diabetes mellitus patients in Yemen. Trop. J. Pharm. Res..

[45] Yosef T., Nureye D., Tekalign E. (2021). Poor Glycemic Control and Its Contributing Factors Among Type 2 Diabetes Patients at Adama Hospital Medical College in East Ethiopia. Diabetes Metab. Syndr. Obes. Targets Ther..

[46] Al Johani K.A., Kendall G.E., Snider P.D. (2015). Self-management practices among type 2 diabetes patients attending primary healthcare centres in Medina, Saudi Arabia. EMHJ-East. Mediterr. Health J..

[47] Alkhormi A.H., Alshahrani N.Z., Mahmood S.E. (2021). Khat chewing leads to increase in glycaemic parameters in patients with type 2 diabetes mellitus in Jazan region, Saudi Arabia and Yemen. Diabetes Metab. Syndr. Clin. Res. Rev..

[48] Chlebowy D.O., Batscha C., Kubiak N., Crawford T. (2019). Relationships of depression, anxiety, and stress with adherence to self-management behaviors and diabetes measures in African American adults with type 2 diabetes. J. Racial Ethn. Health Disparities.

[49] Lin K., Park C., Li M., Wang X., Li X., Li W., Quinn L. (2017). Effects of depression, diabetes distress, diabetes self-efficacy, and diabetes self-management on glycemic control among Chinese population with type 2 diabetes mellitus. Diabetes Res. Clin. Pract..

[50] Bickett A., Tapp H. (2016). Anxiety and diabetes: Innovative approaches to management in primary care. Exp. Biol. Med..

